# Flow Dynamics and Mixing in Extracorporeal Support: A Study of the Return Cannula

**DOI:** 10.3389/fbioe.2021.630568

**Published:** 2021-02-10

**Authors:** Julien Lemétayer, L. Mikael Broman, Lisa Prahl Wittberg

**Affiliations:** ^1^FLOW & BioMEx, Department of Engineering Mechanics, Royal Institute of Technology (KTH), Stockholm, Sweden; ^2^ECMO Centre Karolinska, Astrid Lindgren Children's Hospital, Karolinska University Hospital, Stockholm, Sweden; ^3^Department of Physiology and Pharmacology, Karolinska Institutet, Stockholm, Sweden

**Keywords:** ECMO, return cannula, confined jet, mixing, shear stress

## Abstract

Cannulation strategies in medical treatment such as in extracorporeal life support along with the associated cannula position, orientation and design, affects the mixing and the mechanical shear stress appearing in the flow field. This in turn influences platelet activation state and blood cell destruction. In this study, a co-flowing confined jet similar to a return cannula flow configuration found in extracorporeal membrane oxygenation was investigated experimentally. Cannula diameters, flow rate ratios between the jet and the co-flow and cannula position were studied using Particle Image Velocimetry and Planar Laser Induced Fluorescence. The jet was turbulent for all but two cases, in which a transitional regime was observed. The mixing, governed by flow entrainment, shear layer induced vortices and a backflow along the vessel wall, was found to require 9–12 cannula diameters to reach a fully homogeneous mixture. This can be compared to the 22–30 cannula diameters needed to obtain a fully developed flow. Although not significantly affecting mixing characteristics, cannula position altered the development of the flow structures, and hence the shear stress characteristics.

## 1. Introduction

Extracorporeal Membrane Oxygenation (ECMO) is a life-saving treatment used for patient suffering from acute refractory respiratory or cardio-respiratory failure, offering both circulatory and respiratory support. During the COVID-19 pandemic, ECMO has been widely used for cardio-pulmonary support. In Europe only, over 2687 patients (data from January 11) have at this point received ECMO treatment. Although associated with a relatively high survival rate, ECMO is not complication free. Cerebral bleedings and thromboembolism are the most common severe complications leading to death in 80–85% of the cases (Dalton et al., 2015; Fletcher-Sandersjöö et al., 2017). These complications are associated with blood component damage/activation with subsequent increased risks of hemolysis, thrombosis, and emboli (Chen et al., 2019b). Thrombus has been observed in tubing connectors, cannulae, in the artificial lung, as well as in the blood pumps (Fraser et al., 2012; Fujiwara et al., 2017; Hastings et al., 2017). In the complete circuit, venous blood is drained via a drainage cannula from a major vein and then pumped through a membrane lung (artificial lung, oxygenator) where carbon dioxide is cleared from the blood and the blood is re-oxygenated before it is reinfused back into the patient via a return cannula. If the return cannula is placed on the venous side of the patient's circulation veno-venous ECMO (lung support) is offered. If the return is into the arterial side of the circulation veno-arterial ECMO for cardio-pulmonary support is provided. The cannula, pump, and membrane lung are connected with 3/8 inch tubing in the ECMO circuit for the adult. In small children and the newly born the tubing size is of 3/16 or 1/4 inch inner diameter. The recirculation fraction (R_*f*_) (proportion of extracorporeal flow not contributing to the patient's oxygenation) reflects the intrinsic problem associated with veno-venous (VV) ECMO where an immediate drainage of the extracorporeally oxygenated blood reduces the effective flow. Recirculation fraction has no direct influence on complications such as thrombosis. Instead it is indirect, resulting from when the provider increases ECMO blood flow to compensate for R_*f*_, leading to enhanced mixing and higher shear stresses. Recirculation is affected by factors such as the cannula position, orientation, and design (van Heijst et al., 2001; Körver et al., 2012; Palmér et al., 2016). Adjusting the position of the cannulae can lower the recirculation factor from 46% returned oxygenated ECMO blood being directly drained back into the ECMO circuit to as little as 10% (Broman et al., 2015; Palmér et al., 2016). Hence, improving the understanding of mixing properties between oxygenated blood and native blood in the area of return prior to entering the volume from which blood is drained out into the ECMO circuit is of great importance. Moreover, mixing characteristics depends on the flow regime, and directly connected to the flow rates and geometrical dimensions of cannula and vessel.

Regarding thrombus composition, it varies depending on the formation process, where artificial surfaces as well as flow characteristics as regions of high- and low- shear stresses have been identified as locus for platelet activation (Casa and Ku, 2017). Also, the response of platelets to stress is likely to depend on the type of stress (Qiu et al., 2014), i.e., normal and shear stresses. Once activated, platelets may aggregate on artificial surfaces or in low-shear regions binding to fibrin more easily. Also, non-physiological high shear activation induces loss of platelet surface receptors. This may lead to weaker binding to collagen and von Willebrand factor (vWF) at sites of vascular injury where exposure time to high shear stress is a factor. The conditions faced by the platelets leading to activation have been investigated in many studies (see Mains-Balle et al., 2018; van der Meijden and Heemskrek, 2019 for recent reviews). Moreover, mathematical models based on empirical correlations between thrombus growth and shear rate have been developed (Bark et al., 2012; Mehrabadi et al., 2016; Ku et al., 2017). However, the intensity of the shear stress is not the only determinant factor for platelet activation. According to Hellums (1994) and Jesty et al. (2003), platelets can be activated, or even damaged (Giersiepen et al., 1990), through a short exposure to a high shear stress as well as through a long exposure to a moderate shear stress. Yin et al. (2011) also highlighted that a pulsatile low shear stress potentially could lead to enhanced risk for thrombosis. To take account of the shear stress amplitude and the time of exposure to this shear stress, Nobili et al. (2008) proposed Platelet Activation State (PAS) as a parameter to quantify the platelet activation probability. Using this parameter, Fuchs et al. (2018) studied the different components of the ECMO circuit, reporting that the centrifugal pump head presents the highest risk of activation followed by the return cannula. Furthermore, elevated shear stress may also cause damage to the red blood cells (RBCs) inducing hemolysis (Blackshear, 1972). Hemolysis thresholds have been determined by assessing the shear stress levels in a jet (Sallam and Hwang, 1984; Lu et al., 2001), using viscometers (Wurzinger et al., 1985), and in medical pumps (Fraser et al., 2012). As for platelet activation, the exposure time is important in hemolysis as it reduces the shear stress threshold needed to damage the RBCs (Leverett et al., 2018). To quantify the shear stress acting on blood constituents, both viscous shear stress (VSS) and Reynolds shear stress (RSS) have been used (Jones, 1995; Yen et al., 2014).

Although, confined jets have been thoroughly studied in literature (Rajaratnam, 1976; Rehab et al., 1997; Kandakure et al., 2008) and still is a highly active field of research for various applications (Wang et al., 2020; Pathikonda et al., 2021), studies on highly confined jets such as a cannula inserted in a vessel are few. Moreover, commonly the co-flow in previous studies have been larger than the jet flow (for example flame stabilization), and not focusing on a situation where the co-flow is slower than the jet flow as may occur for cannula flows. In terms of numerical simulations of cannula blood flows, early work by Bedingham and Neavin (1991) assessed probable sites for thrombus activation and deposition and De Wachter et al. (1995, 1996) studied shear stress level in dialysis needle. More recently, Grigioni et al. (2002) and Menon et al. (2013) numerically investigated the effect of the geometry of the cannula on blood flow development. However, experimental data of the detailed flow characteristics remain scarce and are challenging due to the both the liquid (blood) and the components to be studied.

In this experimental study, the focus is on return cannula flows relevant for ECMO cannulation in VV ECMO. In VV ECMO, the blood is drained and returned in the inferior and superior vena cava (IVC and SVC, respectively), or *vice versa*, with the right atrium being the connecting volume in which the flow to and from the cannulae are communicating. From a fluid mechanical point of view, this is a highly complex flow case including multiscale physics as well as transient phenomena. Thus, in order to decompose the problem and to separate the effect due to the different variables, we have in this comprehensive study, adopted the approach to systematically study the effect of return cannula position and flow rates in both cannula and vessel, considering a simplified geometry. In our previous study, Lemétayer et al. (2020), the flow structures and velocity field characteristics of a smaller cannula was studied. In this study, the aim was to study the impact of the flow on in particular mixing characteristics and shear stresses. Considering also a larger cannula diameter enabled larger flow rates to be assessed, including a wider range of cannula to vessel flow rate ratios and the effect of change in confinement.

Although ECMO is the clinical application forming the basis of this study, cannula flows in which blood is drained from or returned to the circulatory system is relevant in several medical situations where the geometry of the cannula, its position in the vessel and the flow rates are parameters affecting the flow characteristics and potentially increase in risk of thrombus formation and/or hemolysis.

## 2. Experimental Methods and Materials

### 2.1. Experimental Rig

**Figure 1:**
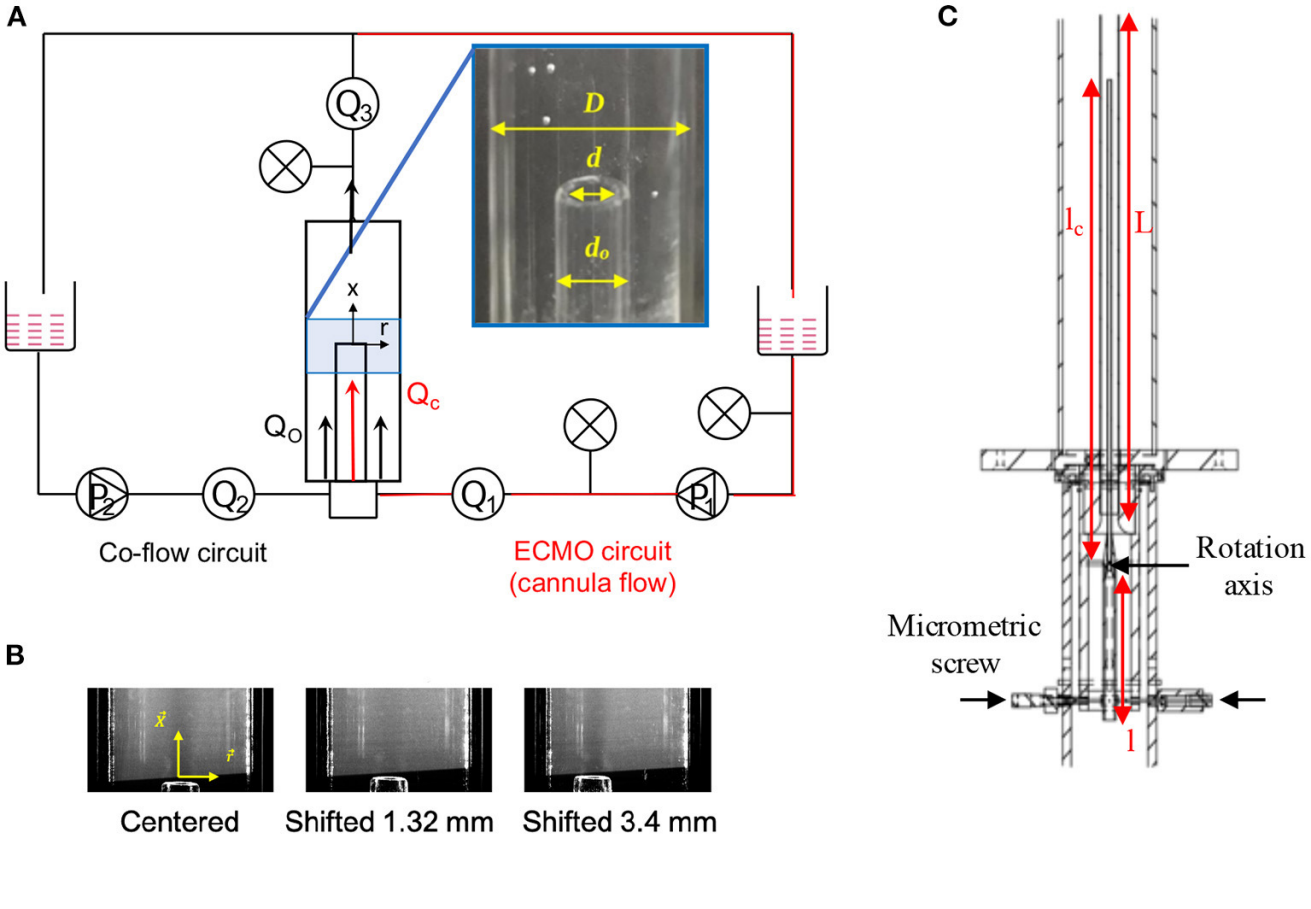
**(A)** Scheme of the experimental rig with the instrumentation including a picture of the cannula inside the outer tube with corresponding dimensions. The co-flow flow circuit is the equivalent of the native venous flow circuit. **(B)** Pictures of cannula alignment for the three different positions studied. **(C)** Drawing of the tube system showing the dimensions of the inlet elements and the inclination system: length tube before constriction *l* = 400 mm, cannula length *l*_*c*_ = 500 mm, and outer tube length *L* = 1 m.

To represent the flow configuration with a cannula in a vessel, two coaxial tubes were used where the outer and inner glass tubes represented the vessel and the cannula, respectively (Figure 1). The outer glass tube had a diameter *D* = 18.3 mm whereas two different cannulae were investigated, a smaller cannula having an inner diameter *d*_*SC*_ = 3.2 mm and an outer diameter *d*_*SC*_*O*__ = 5 mm and a larger cannula having a inner diameter *d*_*LC*_ = 5.5 mm and an outer diameter *d*_*LC*_*O*__ = 7.5 mm. In ECMO, cannulae are available in sizes from 6 to 29 French (Fr, 1 Fr = 1/3× the outer cannula diameter d_*o*_ in millimeters). The cannulae investigated here are similar to the inner diameter of a 13 and 21 Fr cannula, respectively^1^. Regarding the dimensions chosen, the outer tube diameter is in accordance with literature reporting that the IVC mean diameter varies between 18 and 20 mm (Prince et al., 1983; Finnerty et al., 2017). However, the IVC diameter do vary between inhalation and exhalation phases. As shown in Figure 1, two flow circuits were used; one driving the co-flow mimicking the native venous flow and second using a centrifugal blood pump to drive the cannula flow. The 500 mm long cannula was positioned so that the cannula tip (exit) was localized at half height of the outer tube. These lengths were chosen to guarantee a stationary flow in each tube upstream of the cannula tip. To enable studying the flow of a cannula positioned closer to the vessel wall, an inclination system at the base of the experimental set-up (rotation axis on Figure 1C) was used to tilt the cannula relative to the outer tube. However, the dimensions of the tubes limited the tilt angle of the cannula to a maximum of α <0.5°. In the following, the cannula displacement will be referred to as a *lateral shift* with its tip position defined by *r*_*c*_, the distance between the cannula centreline and the outer tube centreline.

The fluid used in the experiments was water with a temperature of (23±2°C). The choice of water was motivated by the strong shear rates expected in cannula flows. For shear rates greater than 100s^−1^, blood viscosity is (almost) Newtonian and about 2–4 times that of water depending on hematocrit (In ECMO hematocrit levels of 20–40% may be used and depends on the providing ECMO center). This in turn makes it possible to scale the flow using the Reynolds number.

To reduce undesired optical deformations due to the curvature of the outer tube, the two glass tubes were surrounded by a rectangular glass box filled with water. Moreover, flow and pressure transducers were positioned at positions as indicated by Figure 1A.

The coordinate system used is defined by the axial axis *x* and the radial axis *r*. The origin of the axes was located where the outer tube centreline intersects the cannula tip cross-section. The notation applied for the mean velocity, instantaneous velocity and the root-mean-square (rms) fluctuations along x-axis are *U*, *u*, and *u*′, respectively. Similarly, *V*, *v*, and *v*′ represent the mean velocity, the instantaneous velocity, and the rms fluctuations along the r-axis.

### 2.2. Measurements Techniques

Two different measurement techniques were used: Particle Image Velocimetry (PIV) and Planar Laser-Induced Fluorescence (PLIF). The interrogation window in which flow characteristics were measured was located in a vertical plane along the cannula axis from the cannula tip up to 60 mm downstream.

For PIV, solid particles of Zirconium oxide having a diameter of 5 μm were used as tracer particles. The PIV system was composed of a high-speed Nd:YAG laser operating at 532 nm, a Dantec high-speed double frame CCD camera (12-bit pixel depth with a sensor 1,920 × 1,200 pix) having a Nikkor 105 mm lens (*f*/*d*_*max*_ = 1.8, Nikon). A 20 mm extension ring was applied to reach a magnification factor of about 31 pix/mm. The laser sheet was formed with a combination of lenses and aligned to pass through the center of the tubes. The laser sheet thickness was 1 mm. For each case, 1,000 double images were acquired at 200 Hz.

To acquire the velocity field from the PIV measurements, an in-house software developed by Lecordier and Trinité (2004) was applied. This method uses a multi-pass algorithm with an original iterative deformation of the particle images to progressively suppress the bias induced by the velocity gradients and improves the calculation at the interface between the jet and the co-flow. The number of spurious vectors never exceeded 1% of the total number of vectors. Calculating the cut-off wavenumber (Foucaut et al., 2004), the spatial resolution of the PIV measurement in the current configuration was 185 μm.

PLIF images were acquired to study mixture characteristics in the same plane as used for PIV. A qualitative measurement was performed at 200 Hz. The system used was similar to that used for PIV, except for an optical filter OG570 added in front of the camera to collect only the fluorescent signal. Due to the non-linearity of the response of the Dantec camera to the fluorescence intensity, the quantitative mixture fraction measurement had to be performed at 5 Hz by a camera Lavision FlowMaster 3S with a Nikkor 50 mm lens (Nikon). For both measurements, the fluorescing tracer was an aqueous solution of Rhodamine B having a concentration of 50 μg/L ejected from the cannula.

For the quantitative measurement of the mixture fraction, a first signal calibration was carried out in a fluorescence cell to determine the dye concentration range yielding a linear dependence of the fluorescence signal with the concentration and the power laser. Afterwards, a second calibration was carried out in the test section by filling the entire volume to validate the first calibration *in situ*. Based on the method described by Balusamy (2010) and adapted from the formulation proposed by Shan et al. (2004), this calibration was used to correct the spatial inhomogeneity of the laser sheet energy and the shot-to-shot variation of the total laser energy. Here, the spatial distribution of energy in the laser sheet was assumed to not change between each laser pulse. The spatial resolution of the PLIF measurement was determined by considering the response of the camera to a step-like transition in the light intensity (Smith, 1997). In the present case, the spatial resolution of the PLIF system was measured at 250 μm from the fluorescence signal transition thickness induced by a sharp obstacle placed in the laser sheet.

Due to the laser reflections on the outer tube and cannula tip and optical deformation due to the outer tube curvature, it was not possible to resolve the velocity field in the near-wall region and close to the cannula tip. Thus, velocity measurement interrogation window started 1 mm downstream of the cannula tip. Also, along the wall, a 1 mm wide region (radial direction) was excluded from the measurements. Further details regarding sensitivity assessment of the experimental setup and measurement technique are found in Lemétayer et al. (2020).

### 2.3. Case Set-Up

Fourteen different cases were studied (Table 1). The same set of measurements were carried out for both cannulae and the parameters changed were cannula flow rate (*Q*_*c*_) and lateral position (*r*_*c*_). To capture a dynamically similar flow as occurring in the IVC for flowing blood, the Reynolds number was used as a scaling factor. Hence, the co-flow (using water as liquid medium) was scaled by keeping the Reynolds number estimated from an expected IVC flow rate and blood viscosity constant. This resulted in a flow rate in the outer tube (*Q*_*o*_, co-flow) of 1.3 L/min, corresponding to a flow velocity of approximately 0.1 m/s. Different flow rate ratios (*Q* = *Q*_*o*_/*Q*_*c*_) were investigated, namely: *Q* = 1, 0.5, 0.33, and 0.25, corresponding to cannula flow rates of 1.3, 2.6, 3.9, and 5.2 L/min, respectively. However, for the smaller cannula only the two larger flow rate ratios (*Q* = 1 and 0.5) were studied. The reason for this was due to the smaller cannula diameter, allowing for a maximum flow rate of 2.6 L/min. The choice of flow rates investigated were based on the return cannula flows used in VV ECMO, commonly between 3.5 and 5 L/min.

**Table 1:** The investigated cases and the associated cannula flow rate (*Q*_*c*_), outer tube (vessel) flow rate (*Q*_*o*_), and cannula position (*r*_*c*_).

**Number case**	**Flow rate cannula *Q*_*c*_ (L/min)**	**Flow rate outer tube *Q*_*o*_ (L/min)**	**Cannula position *r*_*c*_ (mm)**	**Jet velocity $U_{0}^{in}$ (m/s)**	**Reynolds number *Re*_*jet*_**
Case 1_*SC*_	1.3	1.3	0	2.7 (3.2)	8,620
Case 2_*SC*_	2.6	1.3	0	5.4 (6.6)	17,240
Case 3_*SC*_	1.3	1.3	−1.32	2.7 (3.1)	8,620
Case 4_*SC*_	2.6	1.3	−1.32	5.4 (6.3)	17,240
Case 5_*SC*_	1.3	1.3	−3.4	2.7 (3.1)	8,620
Case 6_*SC*_	2.6	1.3	−3.4	5.4 (6.2)	17,240
Case 1_*LC*_	1.3	1.3	0	0.9 (1.1)	5,020
Case 2_*LC*_	2.6	1.3	0	1.8 (2.1)	10,030
Case 3_*LC*_	3.9	1.3	0	2.8 (3.2)	15,050
Case 4_*LC*_	5.2	1.3	0	3.7 (4.3)	20,060
Case 1a_*LC*_	1.17	1.3	0	0.8 (1.1)	4,510
Case 1b_*LC*_	1.43	1.3	0	1 (1.2)	5,520
Case 2a_*LC*_	2.6	1.3	−1.32	1.8 (2.2)	10,030
Case 2b_*LC*_	2.6	1.3	−3.4	1.8 (2.1)	10,030

*For each case, the jet velocity [from $Q_{c}/\left(\pi d^{2}/\text{4}\right)$] and the corresponding Reynolds ($Re=\rho U_{0}^{in}d/\mu$ where d is either d_SC_ or d_LC_ depending on case) is provided. Moreover, in-between brackets, the by PIV measured velocity at the jet axis, i.e., not integrated over the outlet area, is provided*.

From hereon, the following nomenclature will be used: *jet, interface*, and *d* refer to the cannula jet, the interface between the jet and the co-flow, and the inner diameter of either the small or the large cannula (*d*_*SC*_ or *d*_*LC*_), respectively. Moreover, when the cannula is shifted to the left, the left side of the jet will be referred to as the *narrow side* and the right side as the *wide side* (Case 3_*SC*_ to 6_*SC*_).

## 3. Results

To ensure the symmetry of the flow, measurements of the flow velocity and its fluctuations were performed for two symmetric positions of the cannula relative to the outer tube centreline. The results, presented in Lemétayer et al. (2020), showed excellent agreement. Thus, detailed experiments were only carried out tilting the cannula toward one side relative the outer tube centreline.

### 3.1. General Flow Characteristics

**Figure 2:**
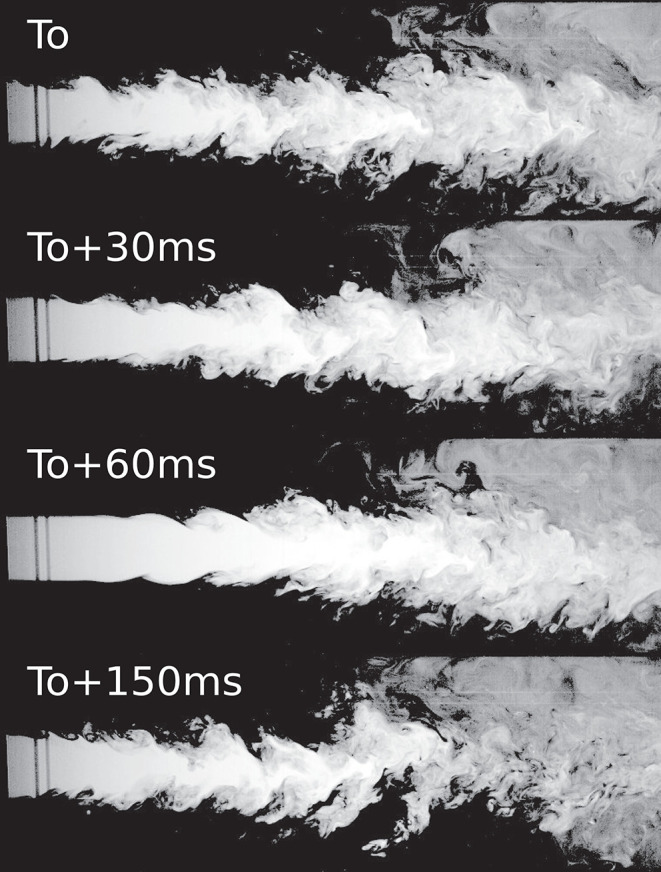
Case 1_*LC*_ for which a transitional flow was observed. The figure shows four consecutive time instances highlighting how the flow went from turbulent to laminar and turning turbulent again.

Except for Case 1_*LC*_, the cannula flows investigated were fully turbulent. The co-flow upstream of the cannula tip on the other hand, remained laminar at all times. For the smaller cannula, the resulting jet was turbulent and characterized by a Reynolds number based on the cannula flow rate *Q*_*c*_ and the cannula inner diameter *d*, corresponding to *Re*_*jet*_ = 8,620 and *Re*_*jet*_ = 17,240 for *Q* = 1 and *Q* = 0.5, respectively. For the larger cannula, the jet was characterized by a Reynolds number ranging from *Re*_*jet*_ = 5,020 to *Re*_*jet*_ = 20,060 for *Q* = 1 and *Q* = 0.25, respectively. For Case 1_*LC*_, a transitional flow regime was observed where the flow altered between laminar, transitioning into turbulent to re-laminarize (Figure 2). As shown in Table 1, the Reynolds number for this case was 5,020. By altering the cannula flow rate by 10%, the flow remained either fully laminar or turbulent for decreasing or increasing flow rate, respectively.

Independent of cannula diameter or flow rate, the velocity fluctuations in the jet were found to be around 5% at *x*/*d* = 1.7, whereas close to zero in the co-flow region. However, as expected, at the interface between the jet and co-flow, the velocity fluctuations were larger, and highly unsteady due to the velocity gradient appearing in the shear layer. For the smaller cannula, the fluctuations were found to be twice that of the large cannula, 20 vs. 10%, respectively. Further downstream, flow unsteadiness was enhanced, reaching rms fluctuation values up to 30 and 50% for the large and small cannula, respectively. For the perturbations introduced by the jet to fully dissipate, 30 and 22 cannula diameters were required for the small and larger cannula, respectively (although the corresponding absolute distance increased with cannula diameter).

**Figure 3:**
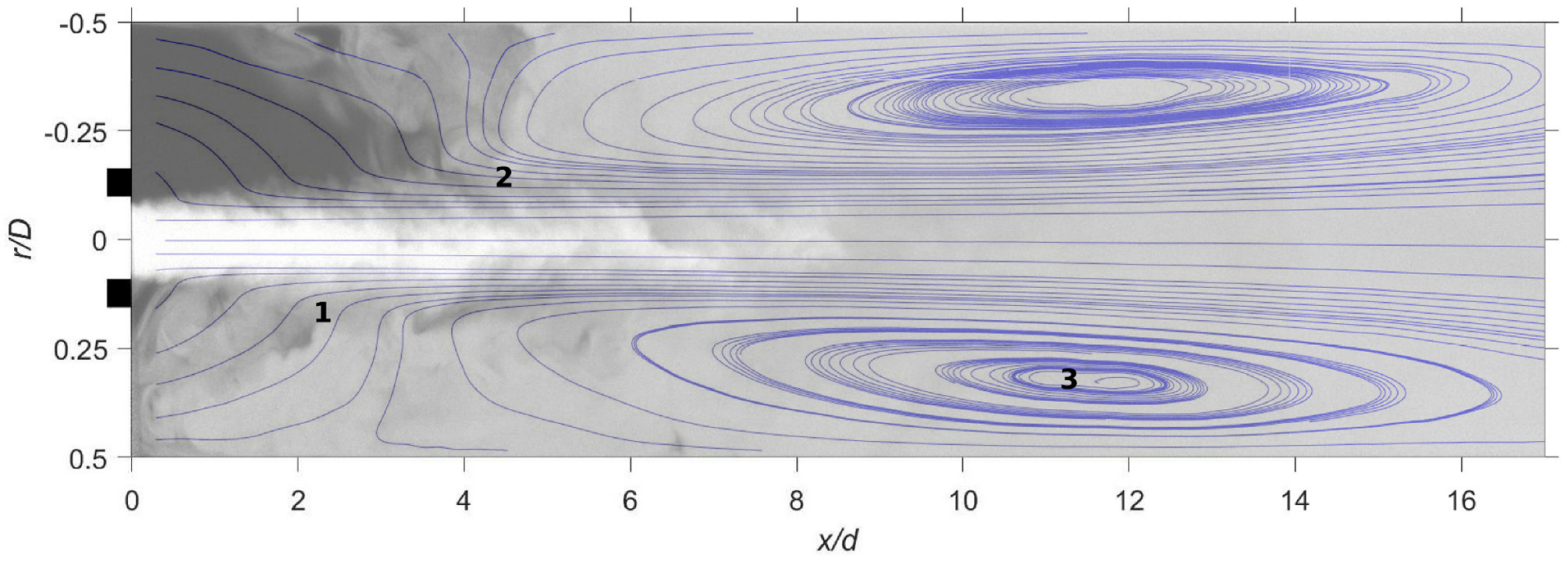
Streamlines for Case 2_*SC*_ (*Q* = 0.5 and *r*_*c*_ = 0) superimposed with an instantaneous scalar field of the mixture fraction. The black lines at the y-axis indicates cannula outlet position.

**Figure 4:**
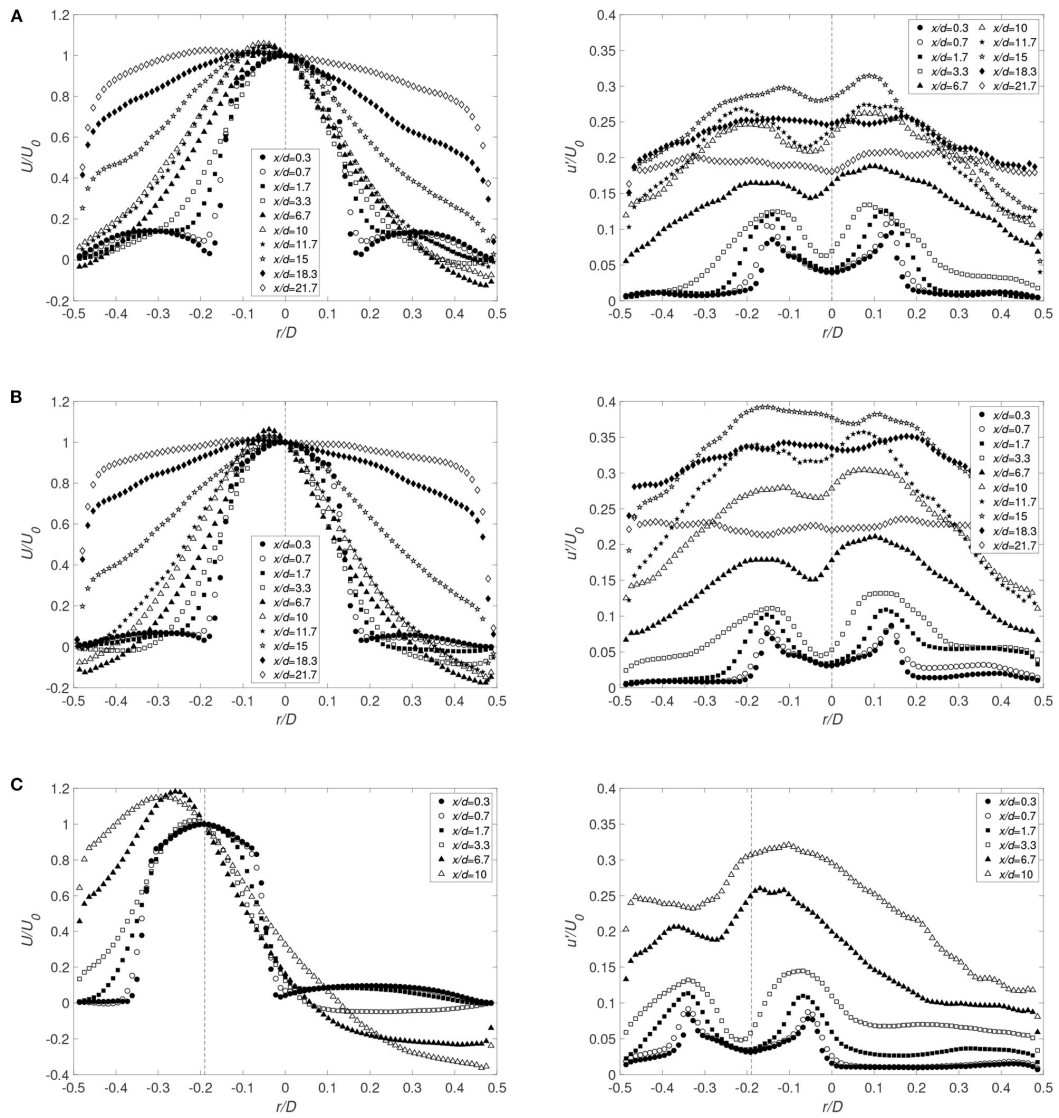
Radial distributions of the averaged streamwise velocity component and corresponding rms fluctuations for the large cannula for **(A)** *Q* = 1 and *r*_*c*_ = 0 (Case 1_*LC*_), **(B)** *Q* = 0.5 and *r*_*c*_ = 0 (Case 2_*LC*_), and **(C)** *Q* = 0.5 and *r*_*c*_ = −3.4 mm (Case 2b_*LC*_). *x*/*d* indicates the distance from the cannula tip normalized by cannula diameter (*U*_0_ corresponds to the averaged value of the streamwise velocity on the cannula axis). Cannula axis is represented by the dashed line.

As presented in our previous work (Lemétayer et al., 2020), three main flow characteristics govern cannula flows characteristics (indicated in Figure 3); lateral entrainment (1), jet vortices (2), and recirculation zones (3) where also a larger-scale backflow motion developed. Obviously, this also applies for the larger cannula investigated here. Moreover, a lateral displacement of the cannula modifies the flow structures, altering the mixing characteristics and the residence times. On the narrow side, vortices cannot grow due to the proximity of the outer wall in which the recirculation disappears. The recirculation zone on the wide side is also altered, expanding in size and increasing the recirculating flow rate, displayed in Figure 4C via the negative streamwise velocity (here for the large cannula). As expected, a jet velocity at the cannula tip faster than the co-flow leads to entrainment of the co-flow by and into the jet (*x*/*d* = 1.7, Figures 4A,B). For the same *Q*, an increase in cannula diameter obviously results in lower cannula velocity and consequently a smaller relative velocity between cannula and co-flow. This in turn results in an entrainment radial velocity about three times lower than that of the small cannula case. Moreover, when tilted, the velocity fluctuations showed that the shear layer on the narrow side vanished earlier as compared to the central cannula configuration (Figure 4, *x*/*d* ≥ 6.7). Consequently, it can be hypothesized that as a larger shear layer region develops when *r*_*c*_ = 0, a cannula positioned close to a vessel wall may expose the blood components to lower shear stress.

**Figure 5:**
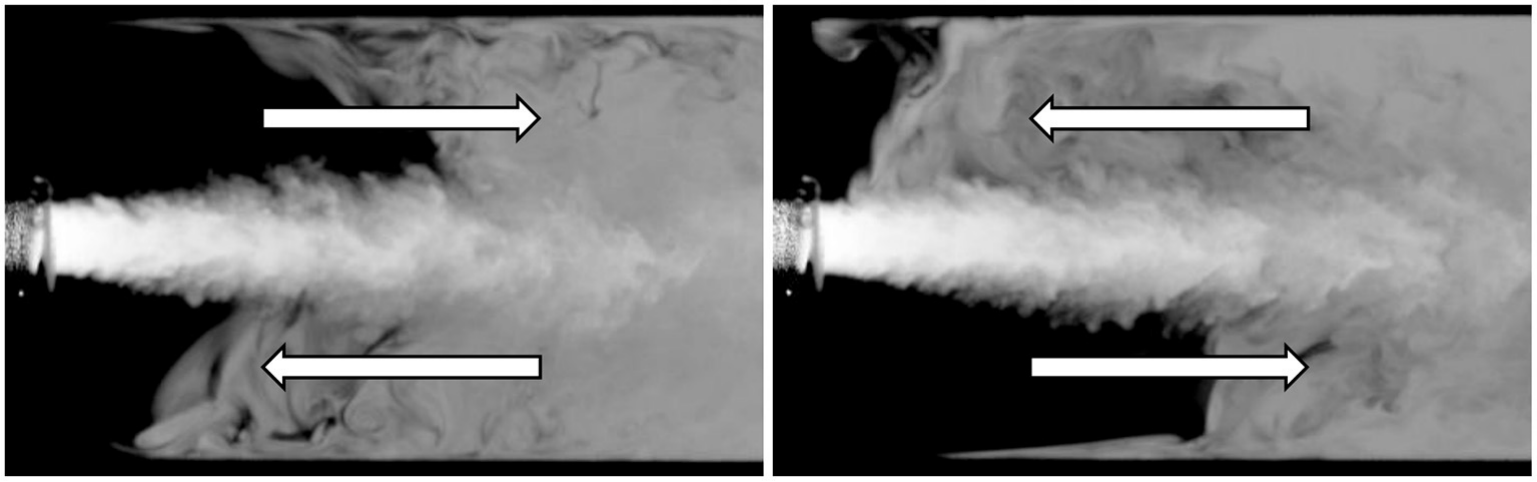
Instantaneous flow field of the mixture fraction for the Case 1_*SC*_ with a 1 s interval.

Due to the confinement imposed by the outer tube, a backflow highly sensitive to modifications in cannula position and flow rate ratio developed. Figure 5 presents the instantaneous flow patterns of the mixture fraction field at two different times. The presence of a backflow on each side of the jet is displayed with the two opposite flow directions highlighting its oscillatory nature. The origin of the backflow is found at a downstream location of similar absolute distance from the cannula tip, corresponding to around *x*/*d* = 18 and *x*/*d* = 10 − 11 for the small and large cannula diameter, respectively. At this location, a separation point in the near-wall flow was found, splitting it into two streams: one moving downstream and another moving back upstream to form the backflow. Fast Fourier Transformation (FFT) of the streamwise velocity fluctuations carried out for the small cannula showed a main frequency around 2 and 4 Hz for *Q* = 1 and 0.5, respectively. For the big cannula, the backflow frequency was found similar to that of the small cannula case, ranging from approximately 1–5 Hz depending of *Q*, with increasing frequency with decreasing *Q*. Moreover, the main pulsation frequency remained close to constant independently of the cannula position for a specified flow rate. Thus, cannula flow has a larger influence on the pulsation frequency as compared to cannula position.

#### 3.1.1. Possible Similarities

**Figure 6:**
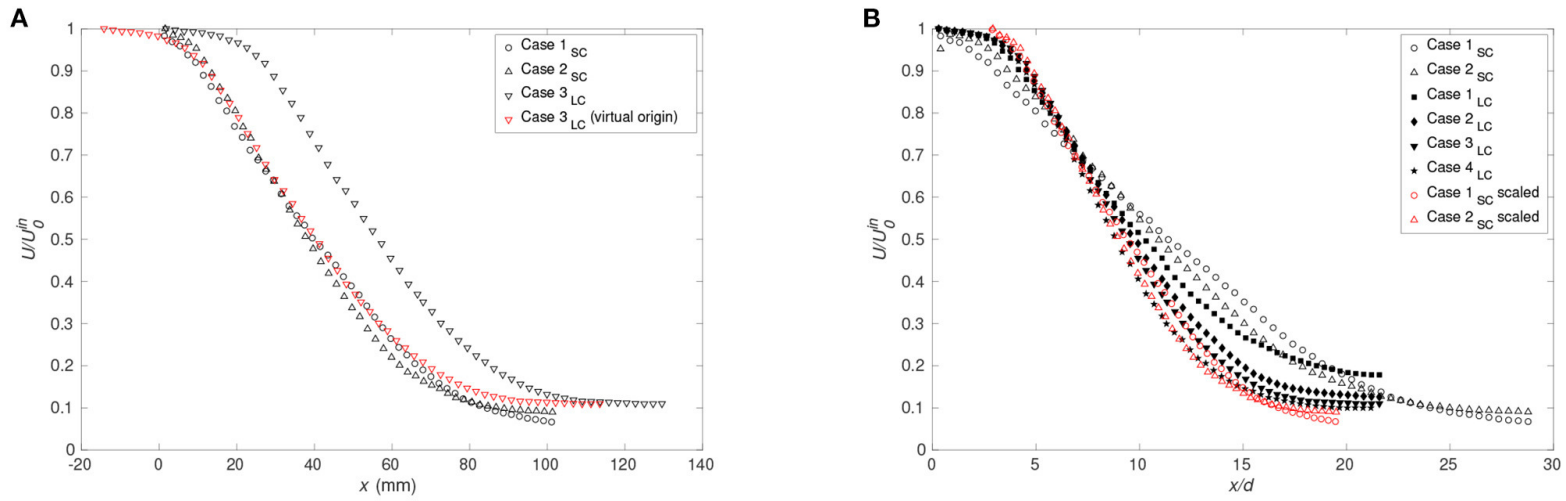
**(A)** Streamwise velocity normalized by inlet jet velocity vs. distance from cannula tip for Case 1_*SC*_, Case 2_*SC*_, Case 3_*LC*_ as well as Case 3_*LC*_ shifted by 16 mm and **(B)** comparison of the decay of the streamwise velocity normalized by the averaged value of the streamwise velocity on the cannula axis near its exit $U_{0}^{in}$ for *r*_*c*_ = 0 for the small cannula (Case 1_*SC*_ and 2_*SC*_) and for the large cannula (Case 1_*LC*_ to 4_*LC*_). The red symbols correspond to the streamwise velocity for Cases 1_*SC*_ and 2_*SC*_ applying the function: (*x* + 16)/*d*_*SC*_ × *d*_*SC*_/*d*_*LC*_.

Considering the velocity magnitude estimated from the volume flow in co-flow and cannula flow, Case 1_*SC*_ and Case 3_*LC*_ are characterized by similar values; approximately 0.1 and 3.2 m/s for the co-flow and cannula, respectively. Figure 6A displays the axial velocity decay normalized by the jet velocity according to Table 1 along the streamwise direction. By shifting the curve of the large cannula by 16 mm, it overlaps that of the small cannula. This is in line with the characteristics of self-similarity of co-flowing jets and localization of a virtual origin (Uddin and Pollard, 2007). Moreover, this also pinpoints the question regarding appropriate scaling factor(s) enabling capturing dynamic similarity. For instance, considering the initial phase of the streamwise velocity decay, similarity can be achieved if applying a scaling to Cases 1_*SC*_ and 2_*SC*_ based on the cannula diameter ratio [(*x* + 16)/*d*_*SC*_ × *d*_*SC*_/*d*_*LC*_], presented in Figure 6B. It can be noted that *D* − (*d*_*LC*_ − *d*_*SC*_)= 16 mm.

**Figure 7:**
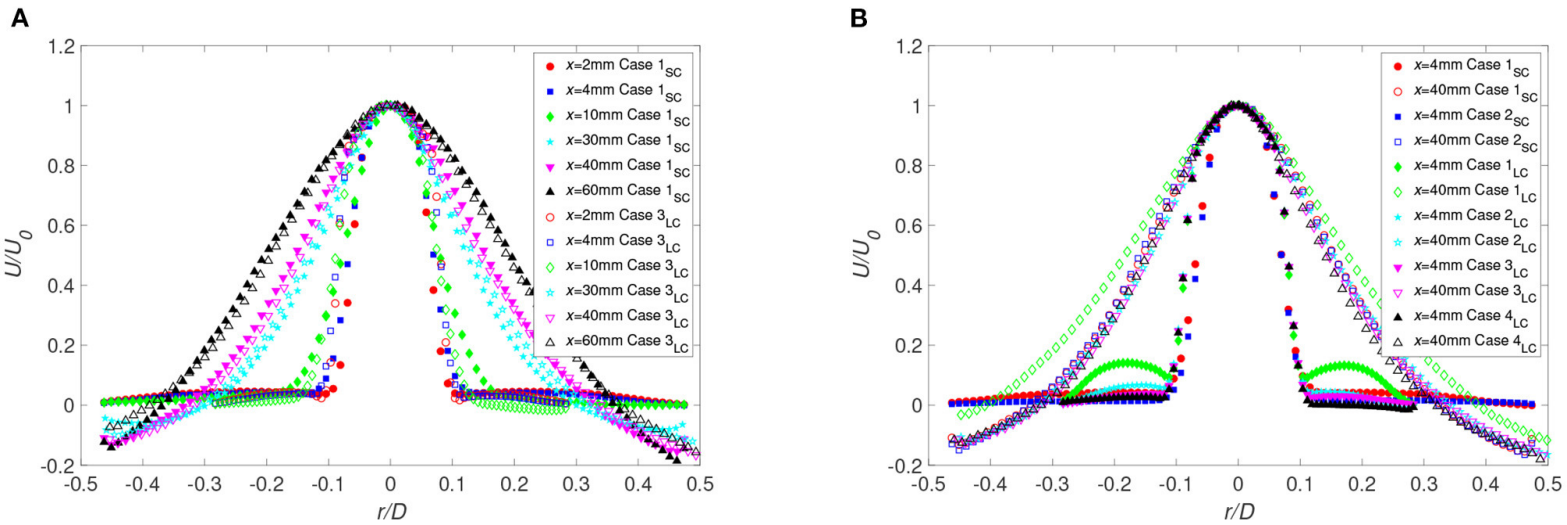
Radial distributions of the averaged streamwise velocity for **(A)** Case 1_*SC*_ and Case 3_*LC*_ and **(B)** all cases with *r*_*c*_ = 0 at different distances from the cannula. For the large cannula cases, the radial position of profiles for *x* ≤ 10 mm is scaled by the cannula diameter ratio *d*_*SC*_/*d*_*LC*_.

Moreover, as for a free jet, similarity of the radial profiles of the streamwise velocity can be achieved by a scaling factor. Figure 7A, presenting the radial profiles at different downstream locations for Cases 1_*SC*_ and 3_*LC*_, shows that similarity exists between cases with similar initial velocities having different cannula diameters. For *x* ≤ 10 mm, similarity is achieved by scaling with the cannula diameter ratio (*d*_*SC*_/*d*_*LC*_) and by the centreline velocity, whereas for *x* ≥ 30 mm, the confinement suppresses the dependency to the cannula diameter ratio. Between *x* = 10 mm and *x* = 30 mm, a scaling factor of the type α*d_*SC*_/*d*_*LC*_, with 1 ≤ α(*x*) ≤ *d*_*LC*_/*d*_*SC*_, proportional to the cannula diameter ratio and function of the downstream location, can be used to collapse the velocity profiles. For different *Q*, Figure 7B, the velocity profiles were found to match well although not to the same extent as for the cases with similar initial velocities. Moreover, Case 1_*LC*_ displays a sensibly different profile far downstream compared to all other cases investigated. This is due to the transitional flow regime characterizing this case, indicating that the flow similarity is solely valid for a turbulent cannula flow.

### 3.2. Mixing Characteristics

As the oxygenated blood is returned to the circulatory system in ECMO, it is important to understand the mixing properties of the oxygen-rich return flow and the oxygen-poor native flow.

**Figure 8:**
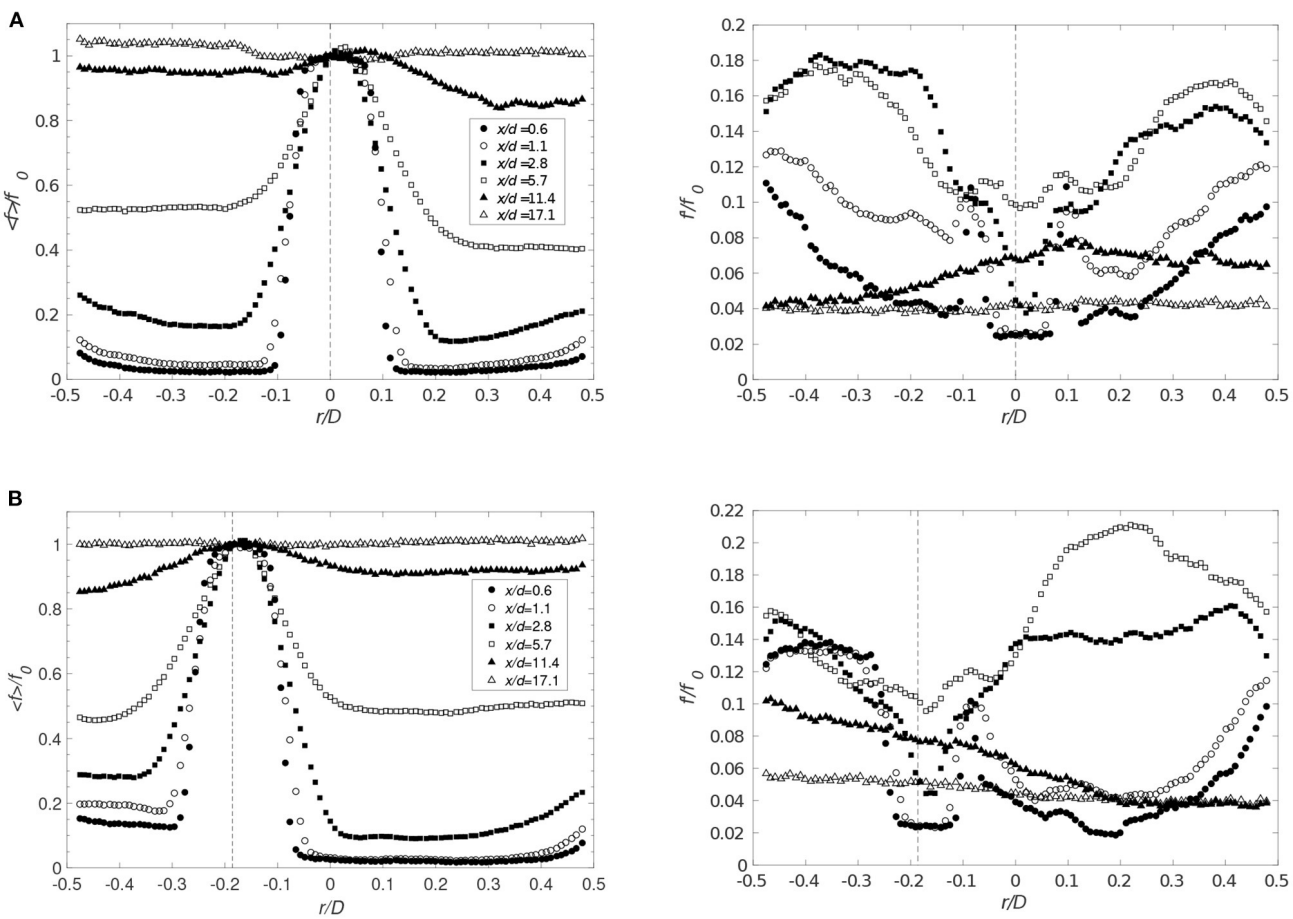
Radial distributions of the averaged values of the mixture fraction (left) and corresponding rms (right) for *Q* = 1 for **(A)** Case 1_*SC*_ (*r*_*c*_ = 0) and **(B)** Case 5_*SC*_ (*r*_*c*_ = −3.4 mm) at different distances from the cannula. Markers identical for < *f*> and *f*′. Cannula axis represented by the dashed line.

**Figure 9:**
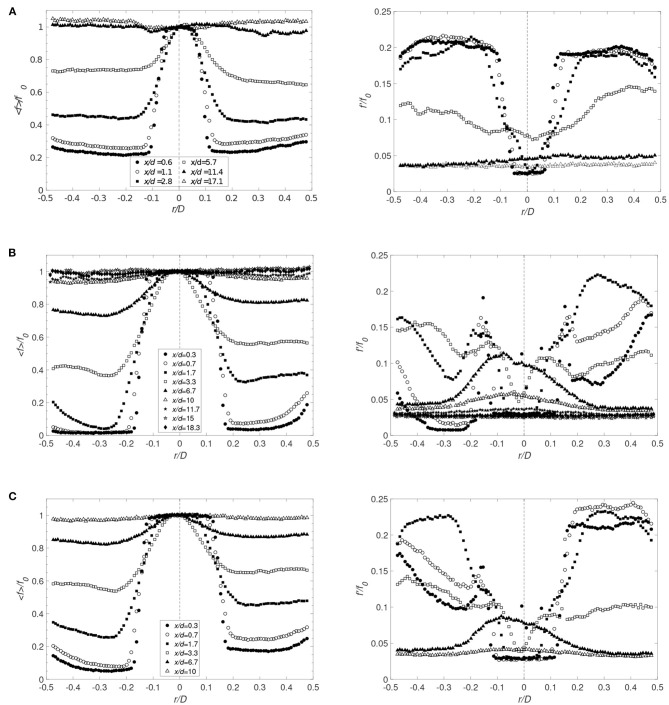
Radial distributions for decreasing *Q* and *r*_*c*_ = 0. The averaged mixture fraction (left figure) and its rms (right figure) are shown for **(A)** Case 2_*SC*_ (*Q* = 0.5), **(B)** Case 2_*LC*_ (*Q* = 0.5), and **(C)** Case 3_*LC*_ (*Q* = 0.33) (*f*_0_ corresponds to the averaged value of the mixture fraction on the cannula axis). Cannula axis is represented by the dashed line.

To asses the mixing process, mixture fraction was used and defined as the local proportion of fluid originating from the jet. A comparison of Figures 4, 8 shows that the radial profiles of the mixture fraction widen faster than the velocity profiles, a feature due to the vortices formed along the interface of the jet. Also, directly at the interface, *x*/*d* ≤ 1.1, theses vortices exhibit strong fluctuations (Figures 8, 9). In the current configuration, the shear layer at the interface and the backflow are the main sources for this rapid mixing. However, the backflow is the most important large-scale structure affecting the flow and the mixing, where the front of the backflow can be characterized by the mixture fraction fluctuations. As observed, significant fluctuations along the wall were found at *x*/*d* = 0.6 up to *x*/*d* = 5.7 (Figure 8A) indicating that the front position of the backflow is highly unsteady. Moreover, for the large cannula, it becomes clear that an increase in cannula flow, decreasing the flow rate ratio cannula to co-flow, moves the backflow and thus the mixing zone upstream, closer to the cannula tip. Low mixture fraction fluctuations are found at *x*/*d* > 10–11.4, confirming that the mixing is complete around that location. Moreover, by further lowering *Q*, the mixture fracture in the large cannula reaches a close to constant profile at *x*/*d* < 10.

A shift in the cannula position had no significant effect on the mean mixture fraction field (Figure 8). However, an increase in mixture fraction fluctuations is observed on the narrow side of the jet at the cross-sections close to the cannula tip, especially for *r*_*c*_ = 3.4 mm. On the other hand, comparing Figure 8A and Figure 9A, a decrease in *Q* considerably influenced the mixing characteristics. This is displayed by an increase in fluctuations on each side of the jet directly downstream of the cannula tip followed by a reduction in fluctuations at *x*/*d* = 5.7, indicating an already well-mixed flow.

#### 3.2.1. Mixing Efficiency

**Figure 10:**
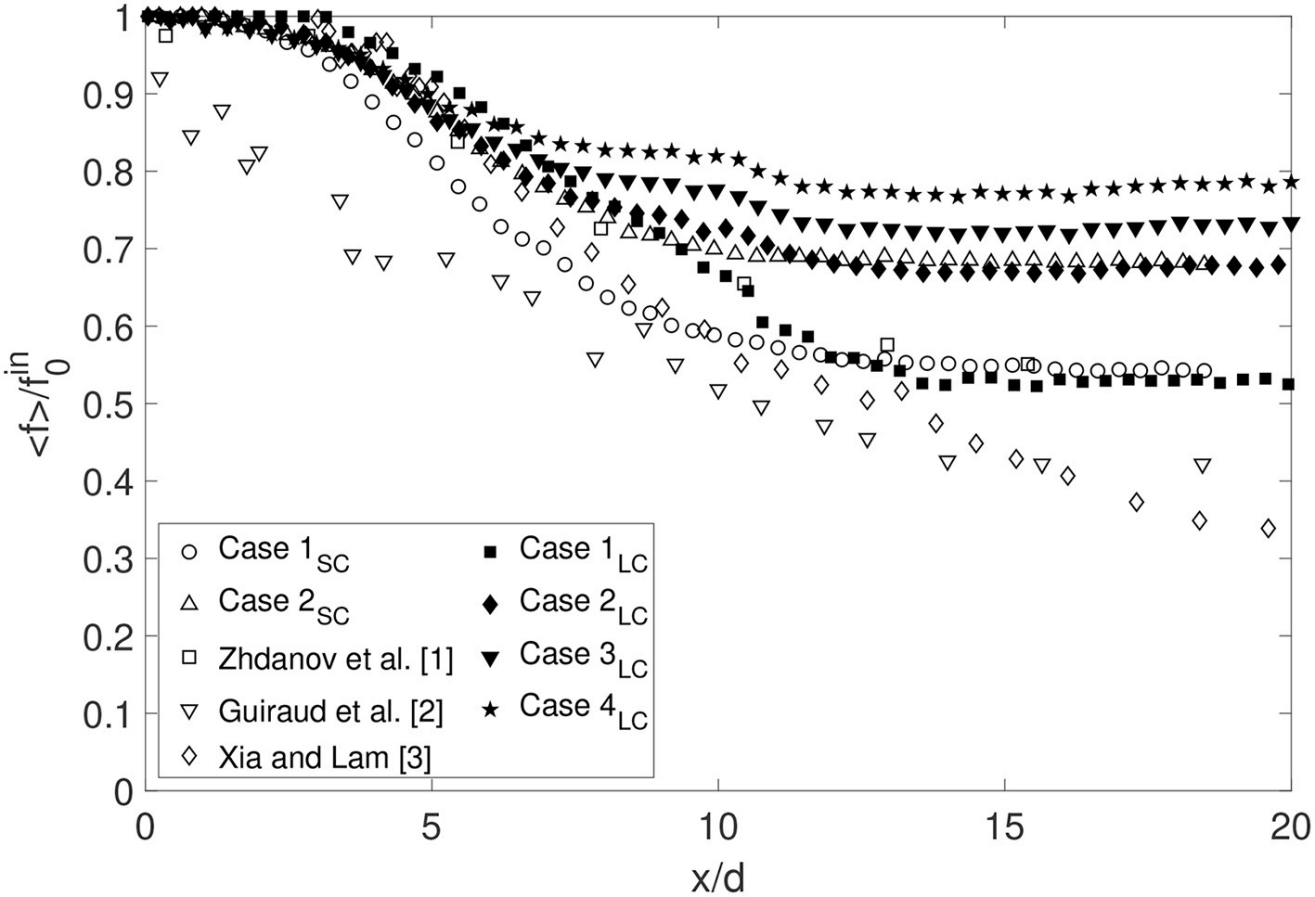
Comparison of the decay of the mixture fraction for *r*_*c*_ = 0 for the small cannula (Case 1_*SC*_ and 2_*SC*_) and for the large cannula (Case 1_*LC*_ to 4_*LC*_) and also including some other studies ($f_{0}^{in}$ corresponds to the averaged value of the mixture fraction on the cannula axis near its exit).

Comparing Figures 4, 8, 9, the radial profile of the mixture fraction approaches a close to constant profile over the cross-section faster than the velocity. Mixing efficiency can be defined as the length required to obtain a homogeneous mixture (i.e., occurrence of minimal or no mixing, characterized by no or very small spatial and/or temporal gradients) across the section of the outer tube. An indication thereof is provided by Figure 10 representing the mixture fraction along the axis. The mixture fraction remains constant from the cannula tip to 1−2 cannula diameter downstream, where the turbulence level remains low (<10 %) avoiding an immediate decrease of the mixture fraction directly at the exit. A similar observation was made in the studies by Zhdanov et al. (2006) and Xia and Lam (2009) having a 5% turbulence level. Furthermore, a comparison with Figure 6 shows that the mixture fraction decay follows the velocity decay until *x*/*d* = 7−9, and is thus controlled by the flow convection. The axial profiles show that the mixture fraction reaches its asymptotic value *f*_*asy*_=1/(1 + *Q*) around *x*/*d* = 9−15. This is considerably faster as compared to the velocity characteristics requiring approximately 20–30 cannula diameters in order to stabilize. This result is in agreement with measurements by Guiraud et al. (1991) showing an asymptotic behavior from *x*/*d* = 14−15 for the same diameter ratio as in Case 1_*SC*_ and Case 2_*SC*_ for a flow rate ratio around 0.7.

**Figure 11:**
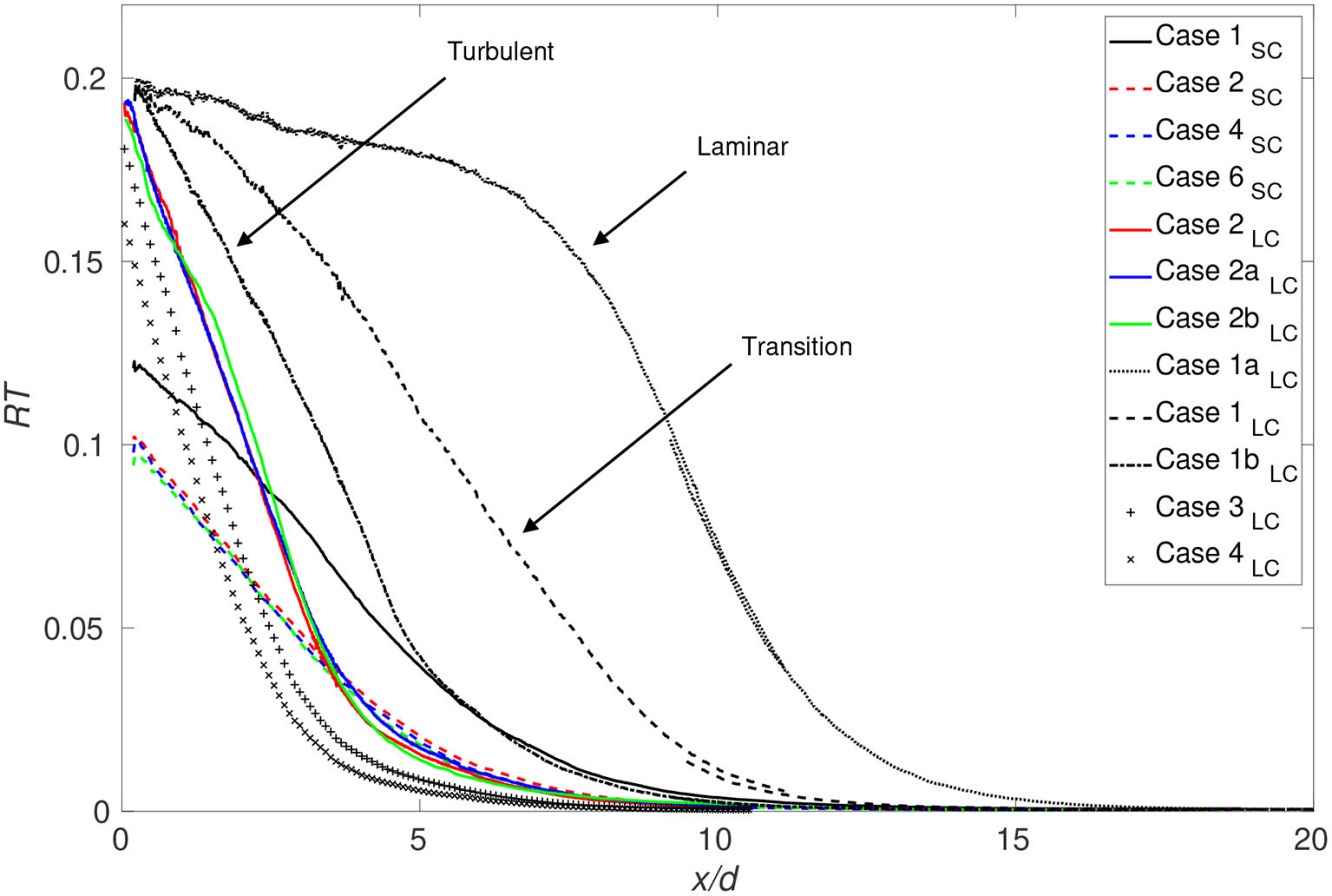
Streamwise evolution of the coefficient *RT* for all cases investigated.

Shifting the position of the cannula affects the flow field as well as the mixing processes. To evaluate to what extent this position modification affects the point at which the flow can be considered homogeneously mixed, the following coefficient *RT* was defined:

(1) $$\begin{array}{l} RT\left(x/d\right)=\frac{1}{T}\int_{0}^{T}R\left(t,x/d\right)dt \end{array}$$

where

(2) $$\begin{array}{l} R\left(t,x/d\right)=\frac{\int {\left(f\left(t,r,x/d\right)-\bar{f\left(t,x/d\right)}\right)}^{2}dr}{D} \end{array}$$

and

(3) $$\begin{array}{l} \bar{f\left(t,x/d\right)}=\frac{\int f\left(t,r,x/d\right)dr}{D} \end{array}$$

where a homogeneous mixture is characterized by a *RT* value approaching zero. Figure 11 shows that although the flow structures change due to the cannula shift, in particular the recirculation, the mixture fraction is homogeneous at a cross-section at the same downstream distance independent of cannula position maintaining the same *Q*. Moreover, decreasing *Q* (faster cannula flow rate), results in a faster mixing. Comparing the small cannula cases with the large cannula cases for the same flow rate ratio, a similar level of mixing is found already at *x*/*d* = 4–5. However, Case 1_*SC*_ and Case 3_*LC*_, having similar initial velocity magnitudes, display differences where Case 3_*LC*_ reaches a homogeneous mixture faster. Recalling the transitional regime observed for Case 1_*LC*_, Figure 11 clearly shows the effect on mixing if the flow is in or close to the transition regime. Although having cannula flow rates only differing by 10%, the onset of mixing is clearly delayed as shown when comparing Cases 1_*LC*_, 1a_*LC*_, and 1b_*LC*_ as well as the length required to reach a homogeneous mixture. For a centered cannula, axisymmetry hypothesis enables calculation of *R* by integrating the mixture fraction over the cross-section resulting in the following equation:

(4) $$\begin{array}{l} R\left(t,x/d\right)=\frac{\int {\left(f\left(t,r,x/d\right)-\bar{f\left(t,x/d\right)}\right)}^{2}2\pi rdr}{\pi D^{2}/4} \end{array}$$

resulting in an initial value and an early behavior different as compared to Equation (2), however, the mixing length remains similar.

### 3.3. Shear Stress

As mentioned in the introduction, quantification of the shear stress in terms of VSS as well as turbulent shear stress is of interest for this flow configuration. VSS is defined as $\tau_{v}=\frac{1}{2}\mu \left(\frac{\partial U_{i}}{\partial x_{j}}+\frac{\partial U_{j}}{\partial x_{i}}\right)$ where the summation convention for tensors holds, so in the present study $\tau_{v}=\mu \left(\frac{\partial U}{\partial y}+\frac{\partial V}{\partial x}\right)$. Considering the turbulent nature of the flow in the current study, the instantaneous stress varies significantly between different instants. Thus, the VSS was assessed for every measurement based on the instantaneous velocity $\tau_{v}=\mu \left(\frac{\partial u}{\partial y}+\frac{\partial v}{\partial x}\right)$. RSS is defined as $-\rho \bar{\left(\text{u'}\text{v'}\right)}$.

The VSS magnitude, governed by the velocity gradient in the shear layer has, as expected, its maximum near the cannula tip and reaches values of about 4 Pa. Moving downstream, the VSS exponentially decreases. Figure 13 presents the RSS fields for both the small and large cannula at different flow rate ratios, showing that RSS is at least one order of magnitude greater than the VSS. Contrary to the VSS, the RSS remains high even far downstream due to large velocity fluctuations found throughout the entire cross-section.

Considering Table 2, a few observations can be made. Firstly, shifting the cannula position appears to lower the VSS, at least in terms of mean values. However, the maximum instantaneous value increases when tilted. Secondly, increasing cannula diameter lowers the VSS, in particular visible for the maximum instantaneous VSS.

**Table 2:** Viscous shear stress characteristics for all cases: spatial maximum on the VSS mean field (1*st* column), maximum VSS over space and time (2*nd* column), VSS standard deviation where the maximum value of the mean VSS is located (3*rd* column), and spatial average of the standard deviation of VSS (4th column).

**Number case**	**Maximum mean VSS (Pa)**	**Maximum instantaneous VSS (Pa)**	**VSS standard deviation at maximum mean VSS location (Pa)**	**VSS mean standard deviation (Pa)**
Case 1_*SC*_	4.21	8.34	1.38	0.16
Case 2_*SC*_	8.32	16.66	4.21	1.02
Case 3_*SC*_	3.88	8.16	1.45	0.17
Case 4_*SC*_	7.3	17.62	8.49	1.38
Case 5_*SC*_	3.64	9.04	2.26	0.19
Case 6_*SC*_	6.94	19.26	8.46	1.38
Case 1_*LC*_	1.39	2.47	0.77	0.14
Case 2_*LC*_	2.32	4.55	1.2	0.25
Case 3_*LC*_	3.38	6.53	1.69	0.38
Case 4_*LC*_	4.55	9.33	2.48	0.52
Case 1a_*LC*_	0.95	2.51	0.14	0.1
Case 1b_*LC*_	1.25	2.5	0.57	0.15
Case 2a_*LC*_	2.60	4.91	1.44	0.3
Case 2b_*LC*_	2.58	5.51	1.42	0.29

**Figure 12:**
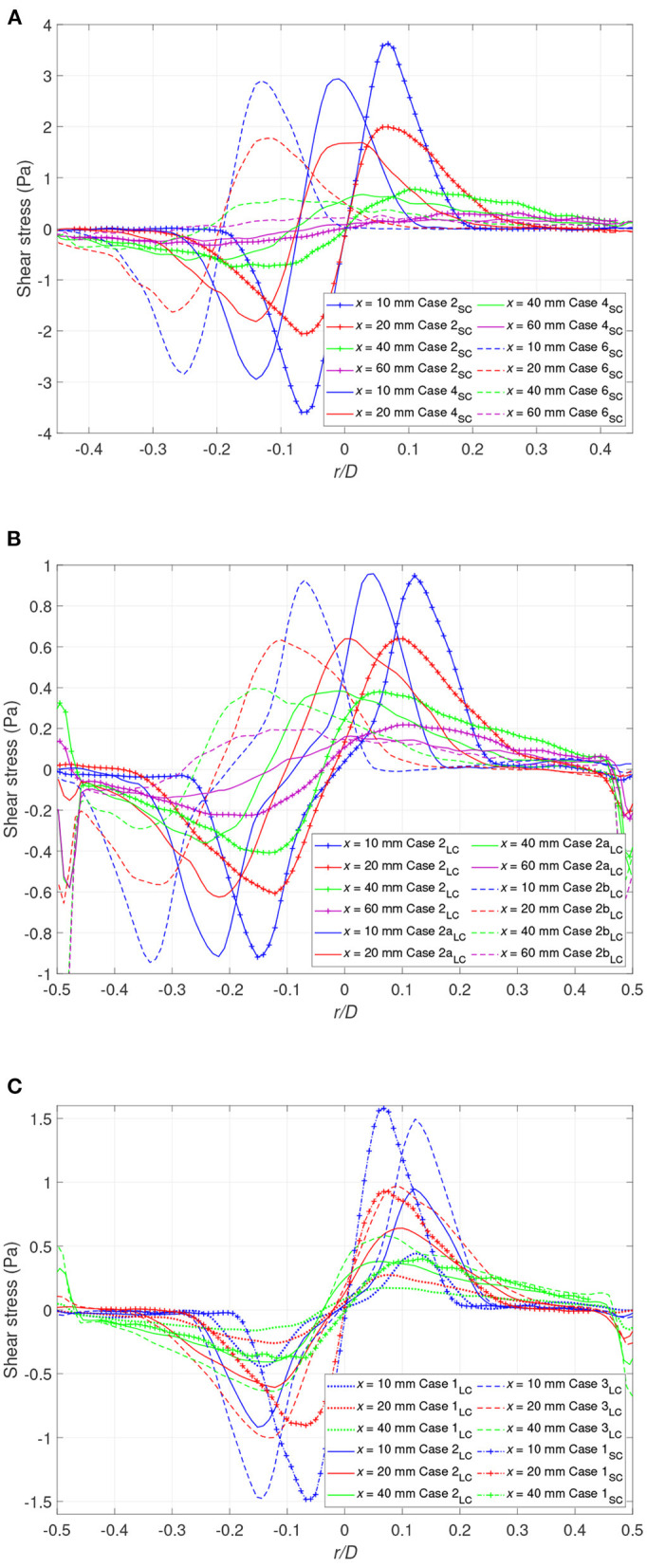
The shear stress profiles at different downstream locations for **(A)** the small cannula and *Q* = 0.5 (Case 2_*SC*_, Case 4_*SC*_, Case 6_*SC*_) **(B)** the large cannula and *Q* = 0.5 (Case 2_*LC*_, Case 2a_*LC*_, Case 2b_*LC*_), and **(C)** the large cannula and *r*_*c*_ = 0 (Case 1_*LC*_, Case 2_*LC*_, Case 3_*LC*_, Case 4_*LC*_), and the small cannula (Case 1_*SC*_).

The velocity profiles in Figure 4 shows that the shear layer dissipated faster when the cannula was tilted toward the vessel wall. This in turn indicated that the shear stresses in this area may be lower as compared to a centrally positioned cannula. This is confirmed in Figure 12, showing the radial distribution of the shear stress at different cross-sections for several flow cases investigated. Close to the cannula tip, there is little difference in-between central or tilted position (Figures 12A,B). However, moving further downstream, the shear stresses on the narrow side decreases with increasing *r*_*c*_. Also, on the wide side of the jet, the shear stresses had similar values independent of to what extent the cannula is tilted for lower jet velocities (Figure 12B). Regarding RSS, it was also affected by the position of the cannula. The more the cannula was shifted, the more the RSS decreased on the narrow side and increased on the wide side, coherent with the dissymmetry observed in the streamwise velocity fluctuations.

**Figure 13:**
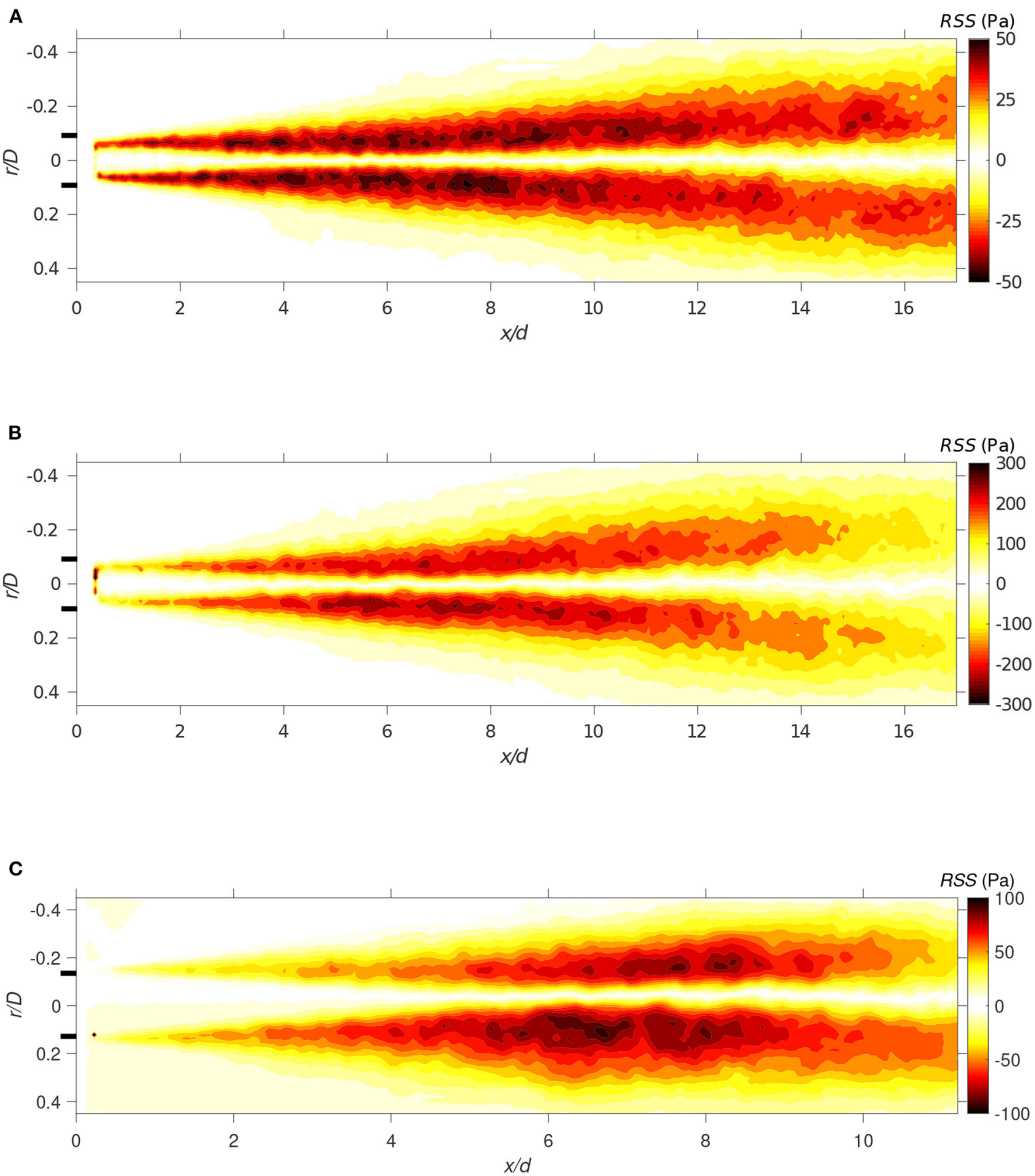
The Reynolds shear stress field for **(A)** Case 1_*SC*_ (*Q* = 1), **(B)** Case 2_*SC*_ (*Q* = 0.5), and **(C)** Case 3_*LC*_ (*Q* = 0.33). All with *r*_*c*_ = 0.

Case 1_*SC*_ and Case 3_*LC*_ have as expected similar levels of shear stress in the shear layer region in close proximity of the cannula tip. However, further downstream this is no longer valid, a consequence of the velocity development, affected by the diameter of the jet core that is twice as large in the large cannula. This feature is also shown by the spatial distribution of RSS in Figure 13, displaying higher values in the downstream region for the large cannula. An increase in cannula flow rate (*Q* ≥ 0.5) results in an increase in shear stresses and the observed increase in RSS is due to a considerable increase in velocity fluctuations along both the axial and radial directions. Thus, due to the high velocity fluctuations appearing in the flow field, RSS is of the same order of magnitude as the thresholds reported to activate platelets and increase risk of hemolysis (Sallam and Hwang, 1984; Hellums, 1994; Fraser et al., 2012; Chen et al., 2019a).

## 4. Discussion and Remarks

Capturing the flow dynamics in a cannula inserted in a vessel is a highly complex problem as there are several variables needed to be taken into account and that varies depending on the clinical situation of interest such as ECMO flows, blood viscosity, position, cannula diameter, and vessel to cannula diameter ratio.

In this work, the research approach was to minimize complexity, in terms of both return cannula design and vessel geometry of the IVC, to enable identifying the underlying fluid dynamical structures influencing cannula performance, in particular in VV ECMO. Shifting the cannula position increases the flow complexity and allows, in a controlled environment, to approach the real situation using flexible cannulae, for which preliminary experiments shows that the cannula is unlikely to be centered in the vessel. Thus, understanding the flow structures involved in and governing mixing in cannula flows and how they are affected by position and operating conditions are important in order to at a later stage provide guidance to the medical community. In terms of geometrical simplification, it especially concerns the outer tube. The diameter used for the outer tube is among the highest values referred for an IVC diameter, meaning that the confinement in a medical situation would likely be more important. However, based on previous studies carried out on confined jets, especially Henzel's work (Henzler, 1978), the flow structures observed in this study would still appear as long as the condition *D*/*d* > *G* + *Q* is valid, where *G* ≈ 1 at equal densities of the mixed fluid. Thus, considering that *Q* should be smaller than 1, the flow structures are expected to remain similar for an outer tube diameter not smaller than twice the cannula inner diameter.

Two different cannula diameters were investigated allowing flow rates from 1.3 to 5.2 L/min to be studied, and as mentioned before, chosen according to the flow rates used in VV ECMO, ranging between 3.5 and 5 L/min although flows of 2–7 L/min are used in clinic and feasible with standard ECMO circuits. Using water in the flow circuit resulted in jet Reynolds numbers of 4,510–20,060 (the incoming co-flow prior cannula tip remained laminar). In all except for the lowest Reynolds numbers, the flow was found to be fully turbulent. However, in the clinical situation, the larger viscosity of blood implies a lower Reynolds number. If estimating a Newtonian blood analog fluid to have 3.4 times the viscosity of water (hematocrit of about 40%), will result in an equivalent blood Reynolds number of about 1,350–4,500 for the considered flow rates. However, in this context, it should be noted that in ECMO, the hematocrit level used by the different ECMO centers varies between 20 and 40%. A hematocrit level of 25% corresponds (at high shear rates) to a viscosity approximately twice that of water. Consequently, the Reynolds numbers obtained scaling with a blood analog Newtonian viscosity suggest that the flow is more likely to laminar to transitional rather than fully turbulent. Estimating the friction factor for a pipe flow using pressure drop measurements of cannula flows using blood (Broman et al., 2019) indicates that the flow is possible to be in transition. Whether the flow is laminar, in transition or turbulent is of clinical importance as the flow regime will alter not only the forces but also the mixing characteristics as shown in Figure 11. As mixing is enhanced by turbulence, the reported mixing lengths required will be larger for a laminar flow. Moreover, if mimicking the flow in the IVC, the flow rate studied is lower than what would be expected. Taking the blood viscosity in account, scaling the flow rates, the higher flow rate ratios (Q = 1 and 0.5) are more relevant from a clinical perspective. It is interesting to note that for the same Q, the small and large cannula perform similarly in terms of mixing length (normalized by diameter) required although initial mixing differentiated. Moreover, comparing Cases 1_*SC*_ and 3_*LC*_ having similar velocities in the cannula jet and co-flow, the large cannula performed better in terms of lowering mixing length required, associated with the larger Reynolds number obtained for the large cannula.

The flow is characterized by strong shear layers and the results show that the shear stresses found in the flow field are of an order comparable to the threshold reported in literature inducing platelet activation or damaging RBCs. This conclusion is not restricted by the use of water. Although using water, we are in our measurements capturing the larger flow scales, also expected to appear in blood. Moreover, even if a reduction of the shear stress magnitude may be expected with blood due to higher viscosity lowering the smallest turbulent scales, it is not only the level of shear stress that the blood constituents are exposed to that is of importance, but also the exposure time (Hellums, 1994; Fraser et al., 2012) that may increase in case of a more viscous fluid. It is commonly accepted that strong shear and long residence times (stagnant regions) are the main contributors for thrombus formation (Casa and Ku, 2017; Chen et al., 2019a). According to Hellums'shear stress vs. exposure time threshold map, the time associated with the shear stresses in the current study (approximately 10^−1^ s) indicates that shear stress levels of the order of 100 Pa are sufficient to activate platelets. In this study, we chose to use two different formulations expressing the shear stress depending on their origin (TVSS and RSS) according to literature. Thus, the formulation used to define the shear stress has an impact on its value since the two formulations result in different orders of magnitude for the shear stress. Furthermore, thresholds measured from a constant shear stress device like viscometer are hardly comparable to the turbulent flow present in the return cannula due to the flow fluctuations making the shear stress varying in space and time. However, studies of real geometries can be used to infer a practical threshold. Liu et al. (1999) found a RSS of 52 Pa studying the flow in an aortic valve and pointed out that this magnitude of RSS is enough to damage RBCs and activate the platelets even if lower than the threshold usually defined. Thus, according to our investigations, the flow created by the reinjection of blood is highly susceptible to present shear stresses activating the platelets or damaging RBCs. However, it should be mentioned that in this study, the shear stress estimation is based on only two components due to the experimental set-up. Thus, the contributions of the third component and its fluctuations are not taken into account, that may lead to an underestimation of the actual shear stresses in the flow.

Shifting the cannula, the results show that the shear stress decreases as compared to the configuration with a centered cannula. On the other hand, the greater fluctuations of the shear stress when the cannula is shifted indicates that higher value of shear stress may be reached locally in the flow. Furthermore, the size of the recirculation zone on the wide side is larger, indicating that there is an elevated risk for platelets to be exposed to an increased residence time in that region. Thus, the presence of structures that can act in order to trap platelets, such as backflow or vortices, has a negative effect on platelet activation by increasing the potential of residing in zones of high shear stress as well during prolonged time. This resembles the observation made by Fuchs et al. (2018) focusing on platelet activation in ECMO circuit components. An increase in cannula flow rate will enhance the shear stress and the backflow characteristics. These results could be considered as an inducement for further developments to improve the control over the cannula position.

From the clinical perspective, the results from this work that directly apply to the clinical situation are (1) increased understanding how distances between the return and drainage cannula relate to R_*f*_ in terms of mixing of the two blood streams, and (2) how cannula size and flow rate, or rather the difference in flow speed of a narrow vs. a wider cannula influences the shear layer (between cannula and native flow) and associated shear forces and thus platelet activation and trauma to the RBCs. With this new knowledge, a clinician can get assistance in choosing the cannula size and better positioning the return cannula in terms of recirculation and blood trauma (platelet activation) with the best interest of the patient in focus.

## 5. Conclusion

In this experimental study of the flow dynamics in a return cannula, the main findings are as follows:

- Flow similarity was found for different cannula diameters having similar jet to co-flow velocity ratio. The scaling was found to change with downstream position *x*. From *x* ≤ 10 mm, the radial position was scaled with the diameter ratio (*d*_*SC*_/*d*_*LC*_) and the velocity with the average value of the streamwise velocity on the cannula axis (*U*_0_). Moving downstream, a scaling factor α needed to be added to the diameter ratio. Also, for the streamwise velocity decay, scaling was found including a shift in streamwise direction combined with the diameter ratio.
- Mixing was controlled by the lateral entrainment at cannula tip, the shear layer and the backflow along the wall. The first two are mainly governed by the flow rate ratio whereas the backflow was also sensitive to cannula position.
- Decreasing co-flow to cannula flow rate ratio (i.e., increasing cannula flow) resulted in a more efficient initial mixing. However, the distance required to obtain a homogeneous mixing was less affected by the flow rate ratio where both cannulae for the same Q resulted in similar mixing lengths.
- The importance of flow regime (laminar, transition, or turbulent jet) and its effect on mixing length was clearly shown, where the laminar flow regime significantly increases the length required to obtain a homogeneous mixture.
- Shifting the cannula toward the wall resulted in a faster dissipation of the shear layer on the narrow side of the cannula. In all cases investigated, the shifted cannula had a smaller area characterized by large velocity fluctuations, that in turn lead to lower shear stresses on the narrow side. On the wide side, the cannula diameter had an effect on the shear stress level. For the small cannula, shear stresses was at close distance to cannula tip affected by cannula position whereas for the large cannula, the shear stresses remained similar.

From the clinical point of view, aiming at preventing recirculation of oxygenated ECMO blood, the results show that a minimum of 10–12 cannula diameters are needed to obtain a homogeneous mixture of oxygenated blood and native blood. As shown, this depends on the flow regime and thus quantifying cannula flows using blood or blood analogs are important, in particular for the larger flow rates and cannula size. Moreover, a fully developed flow was obtained at a much larger distance (22–30 cannula diameters), indicating that higher level of oxygenated blood may be transported over longer distances by the jet. Thus, if placing the drainage cannula too close to this region, this may increase *R*_*f*_, i.e., directly draining oxygenated ECMO blood back into the ECMO circuit.

## Data Availability Statement

The raw data supporting the conclusions of this article will be made available by the authors, without undue reservation.

## Author Contributions

JL performed the experiments, analyzed the results, and drafted the manuscript. LB analyzed the results and critically revised the manuscript for intellectual content. LPW designed the concept, analyzed the results, and critically revised the manuscript for intellectual content. All authors approved for submission.

## Conflict of Interest

The authors declare that the research was conducted in the absence of any commercial or financial relationships that could be construed as a potential conflict of interest.

## Footnotes

^1^The cannula size (diameter) refers to the outer cannula diameter (*d*_*o*_, Figure 1A). The wall thickness is in general 0.39–0.4 mm for cannulae <15 Fr. In lager cannulae the wall thickness is approximately 0.49 mm. The glass tube wall thickness used here is about 0.75 mm. Hence, the inner diameter of the smaller cannula equals a 13 Fr cannula, but *d*_*S*_*C*__*O*__ corresponds to a 15 Fr cannula. For the larger cannula, the corresponding values are 21 and 24 Fr.
